# Neonicotinoid pesticide limits improvement in buzz pollination by bumblebees

**DOI:** 10.1038/s41598-017-14660-x

**Published:** 2017-11-14

**Authors:** P. R. Whitehorn, C. Wallace, M. Vallejo-Marin

**Affiliations:** 1 School of Natural Sciences, University of Stirling, Stirling, FK9 4LA UK; 2 Karlsruhe Institute of Technology (KIT), Institute of Meteorology and Climate Research–Atmospheric Environmental Research (IMK-IFU), 82467 Garmisch-Partenkirchen, Germany

## Abstract

Neonicotinoid pesticides have been linked to global declines of beneficial insects such as bumblebees. Exposure to trace levels of these chemicals causes sub-lethal effects, such as reduced learning and foraging efficiency. Complex behaviours may be particularly vulnerable to the neurotoxic effects of neonicotinoids. Such behaviours may include buzz pollination (sonication), in which pollinators, usually bees, use innate and learned behaviours to generate high-frequency vibrations to release pollen from flowers with specialised anther morphologies. This study assesses the effect of field-realistic, chronic exposure to the widely-used neonicotinoid thiamethoxam on the development of sonication buzz characteristics over time, as well as the collection of pollen from buzz-pollinated flowers. We found that the pollen collection of exposed bees improved less with increasing experience than that of unexposed bees, with exposed bees collecting between 47% and 56% less pollen by the end of 10 trials. We also found evidence of two distinct strategies for maximising pollen collection: (1) extensions to the duration of individual buzzes and (2) extensions of the overall time spent buzzing. We find new complexities in buzz pollination, and conclude that the impacts of field-realistic exposure to a neonicotinoid pesticide may seriously compromise this important ecosystem service.

## Introduction

Understanding the interactions between plants and their insect pollinators is of great practical and political relevance, being vital to efforts to ensure food security under rapid global change^1^. Increased production is often achieved through the deployment of pesticides but this can compromise sustainability by impacting beneficial insect pollinators. This conflict is exemplified by the controversy over neonicotinoid insecticides^2,3^. Neonicotinoids have become the most widely used insecticides in the world^4^ but their use is causing widespread concern, with evidence linking them to the decline of beneficial species such as bumblebees^5,6^. Beneficial insects can be exposed to these insecticides when they forage on flowering crops, as well as wild plants growing on agricultural land^7–9^. This exposure to trace levels of neonicotinoids in nectar and pollen causes a range of sub-lethal effects in bees, such as reduced foraging efficiency^10–12^, impaired navigation^13^, a reduction in learning and memory^14^ and reduced reproductive success^15,16^. These impacts may have substantial impacts at the population level^5,6,17^ and therefore adversely affect the plants that rely on insects for pollination^18^.

A type of pollination that may be particularly sensitive to impairment in learning and memory, due to exposure to pesticides, is buzz pollination^19^. Buzz pollination is a relatively complex biotic interaction, in which pollinators, usually bees, interact with flowers with specialised anther morphologies that require high frequency vibrations to release pollen^20–24^. Buzz pollination has evolved independently many times^25^ and now occurs in approximately 20,000 plant species, including some of the world’s most important crops, such as tomatoes and potatoes^22^. During buzz-pollination, bees grab the anthers with their mandibles, curl their body around the anther cone and then, decoupling their wings from the indirect flight mechanism, they rapidly contract their thoracic muscles, which produces a vibration without the wings beating^21,26,27^. These vibrations, also called buzzes or sonications due to the audible sound they incidentally produce, are transmitted from the bee’s body to the anthers, causing the pollen grains to be released on to the bee, where they can be collected^24^. Previous work has shown that sonication has both innate and learned components^28–30^. These studies showed that naïve foragers are able to effectively sonicate on their first visit to a flower, showing the innateness of the behaviour. However, after a number of visits, the characteristics of the buzzes change. For instance, Morgan *et al*.^29^ found that the peak frequency of sonication declined with experience, and Russell *et al*.^30^ established that the length and amplitude of buzzes increased with experience, suggesting a learned component. It is important to note that neither of these previous studies determined experimentally whether change in sonication characteristics during learning affect the quantity of pollen that bees can remove from flowers. Therefore, to date, we do not know whether sonication learning is associated with increased pollen collection. The challenge of manipulating morphologically complex flowers through the deployment of a multifaceted behaviour potentially makes buzz pollination particularly sensitive to neurotoxic pesticides, such as neonicotinoids.

We know very little about how the ability of bees to buzz-pollinate flowers may be affected by pesticide exposure. In the only study on this to date, Switzer and Combes^19^ looked at buzz pollination behaviour before and after an acute dose of the neonicotinoid imidacloprid. The authors found that the lowest dose of 0.0515 ng did not result in any quantitative changes in the frequency and length of sonication buzzes of *Bombus impatiens* workers. Unfortunately, too few bees exposed to the higher doses (0.515 and 5.15 ng) resumed foraging, and it was therefore not possible to assess how these concentrations impact buzz pollination. Furthermore, the quantity of pollen collected by bees in the different treatments was not assessed. Therefore, the effects of field-realistic, chronic exposure to neonicotinoids on buzz pollination remains to be determined. The present study explores this highly topical subject and aims to determine the effect of the widely-used neonicotinoid thiamethoxam on (1) the characteristics of sonication buzzes and changes over time and (2) the collection of pollen from buzz-pollinated flowers.

## Materials and Methods

Two commercial *Bombus terrestris audax* colonies were obtained from Biobest (Belgium) via Agralan Ltd (Swindon, UK); the first on 8^th^ June 2016 and the second on 20^th^ July 2016. The experiment was carried out with bumblebees from the first colony and then repeated with bumblebees from the second colony. On arrival the majority of workers from the colony were removed and randomly split into three groups, ensuring an approximately equal distribution of worker sizes in each group. These groups of workers were then placed in separate plastic nest boxes (27 cm × 25 cm × 14 cm) where they established queenless microcolonies. Each microcolony was supplied with either control nectar or nectar contaminated with the neonicotinoid thiamethoxam at two concentrations. All groups were provided *ad libitum* with untreated pollen (Biobest via Agralan Ltd).

### Thiamethoxam dilutions

An initial stock solution (10^5^ µg thiamethoxam L^−1^) was made by diluting 10 mg pure thiamethoxam (Sigma-aldrich, UK) in 100 ml acetone. This was further diluted with purified water to 10^4^ µg thiamethoxam L^−1^. Aliquots of the diluted stock solution were added to sucrose solution (Biogluc, Biobest via Agralan ltd) to create concentrations of 2 parts per billion (ppb) and 10 ppb. An equivalent volume of acetone in purified water was added to the control sucrose solution. The concentrations used were chosen to reflect the range of values found in the nectar and pollen of crop and wild plants in the field^7,9,31,32^.

### Pre-treatment and training

The microcolonies were exposed to the treated or control nectar for nine days before the buzz pollination trials began. During this period, the microcolonies were connected to a flight arena for training to encourage workers to leave their nest boxes to forage. The flight arena was a 100 cm × 60 cm × 35 cm wooden box with a Perspex lid. The microcolonies were connected one at a time, each for a total of 14 hours. During this period the arena contained training flowers (non-poricidal *Chrysanthemum* sp.).

### Buzz pollination trials

After the training period, the micro-colonies were connected to the flight arena on a rotational basis, continuously for five weeks (each colony was connected for approximately four hours in either the morning or the afternoon and all three colonies were sequentially connected in a 1.5 day period, allowing each colony to alternate between morning and afternoon sessions). When a microcolony was connected, bees were individually allowed into the flight arena for a ten minute period; a sliding metal door in the connecting tube was used to control bee entry to the arena. A microphone recorder (Zoom H4n Handy Recorder) was set up next to a mesh-covered window in the side of the arena and the entire ten minute session was recorded. Before a bee entered the arena, three *Solanum rostratum* (Solanaceae) flowers were attached to a vertical stick, which was placed next to the mesh window, exactly 5 cm from the recorder. After a bee’s first visit to the arena, she was caught and tagged with a numbered, coloured disc (Opalith, Christian Graze KG, Germany).

Numbered bees were allowed to visit again until they had successfully completed ten arena visits in each of which they buzz pollinated one or more flowers, after which they were not allowed to return. Typically, in a successful foraging bout, an individual bee buzz pollinated all three flowers multiple times. If a bee successfully foraged from the flowers the pollen from her left rear leg was collected at the end of the arena session and stored in a 1.5 ml Eppendorf tube with 1 ml of 70% ethanol. This pollen was later counted using a haemocytometer – the number of grains in 0.1 µl was counted and the number of grains in this aliquot was used for statistical analysis. Three new flowers were then placed in the arena for the next foraging visit.

### Sound analysis

The sound data were analysed using the software Audacity 2.1.2 (www.audacityteam.org). For each trial, a high pass filter with a roll-off of 12 dB per octave and a cut-off frequency of 100 Hz was used to reduce background noise. Spectrograms were used to identify the peak frequency (Hz) and corresponding amplitude (dB) for the first five clear sonication buzzes and the first five clear flight buzzes in each trial. A sonication buzz was defined as a sonication made on the anther of the flower, including all buzz sounds with less than one second pause between them. Buzz sounds that occurred after more than a one second pause (or were separated by a flight buzz) were classified as separate sonication buzzes. The number and duration of sonication buzzes during the first two minutes of sonication were also recorded, from which we calculated the duration of buzzing per minute for each trial. This was then multiplied by the total time the bee spent visiting (the total time in the arena minus the time to when the bee first sonicated) to arrive at an index of buzzing effort. This gives an estimate of the time spent sonicating during the trial.

### Statistical analysis

Data were analysed in R, version 3.1.2. (2014 The R Foundation for Statistical Computing) and were subsetted to only include bees that had completed four or more trials. Preliminary analyses showed that the two colonies behaved differently and so were analysed separately. A generalized linear mixed effect model with a Poisson distribution (with log link function) was used to analyse factors affecting the quantity of pollen collected (i.e. the number of grains). The buzz effort index, buzz duration (Box-Cox transformed to fulfil normality assumptions) and the peak frequency and amplitude of the pollination and flight buzzes were analysed with linear mixed effect models. All models were run in the lme4 package^33^ and individual trial was the unit of replication, with treatment (a factor with three levels), trial number (a covariate from 1 up to 10) and their interaction as fixed effects and the individual bee IDs as a random effect. The significance of fixed effects and their interactions were tested using likelihood ratio tests to compare models with and without the term of interest. 95% CIs were calculated as +/−1.96*SE of the mean.

### Data availability

The datasets generated during the current study are available from the corresponding author on reasonable request.

## Results

A total of 72 bees were observed carrying out 463 arena visits. 44 of these bees carried out at least four arena visits and were included in the analyses (Table 1).

**Table 1:** Summary of the sample size of workers from each colony.

Colony	Treatment	Total no. foragers	Mean & range (min-max) trials completed	No. bees completing 10 trials	No. bees completing > 4 trials.
1	Control	17	4.9 (1–10)	7	7
1	2ppb	13	5.9 (1–10)	7	7
1	10ppb	12	5.5 (1–10)	6	6
2	Control	9	8.2 (1–10	6	8
2	2ppb	10	7.8 (2–10)	6	8
2	10ppb	11	7.6 (1–10)	8	8

### Pollen collection

**Figure 1:**
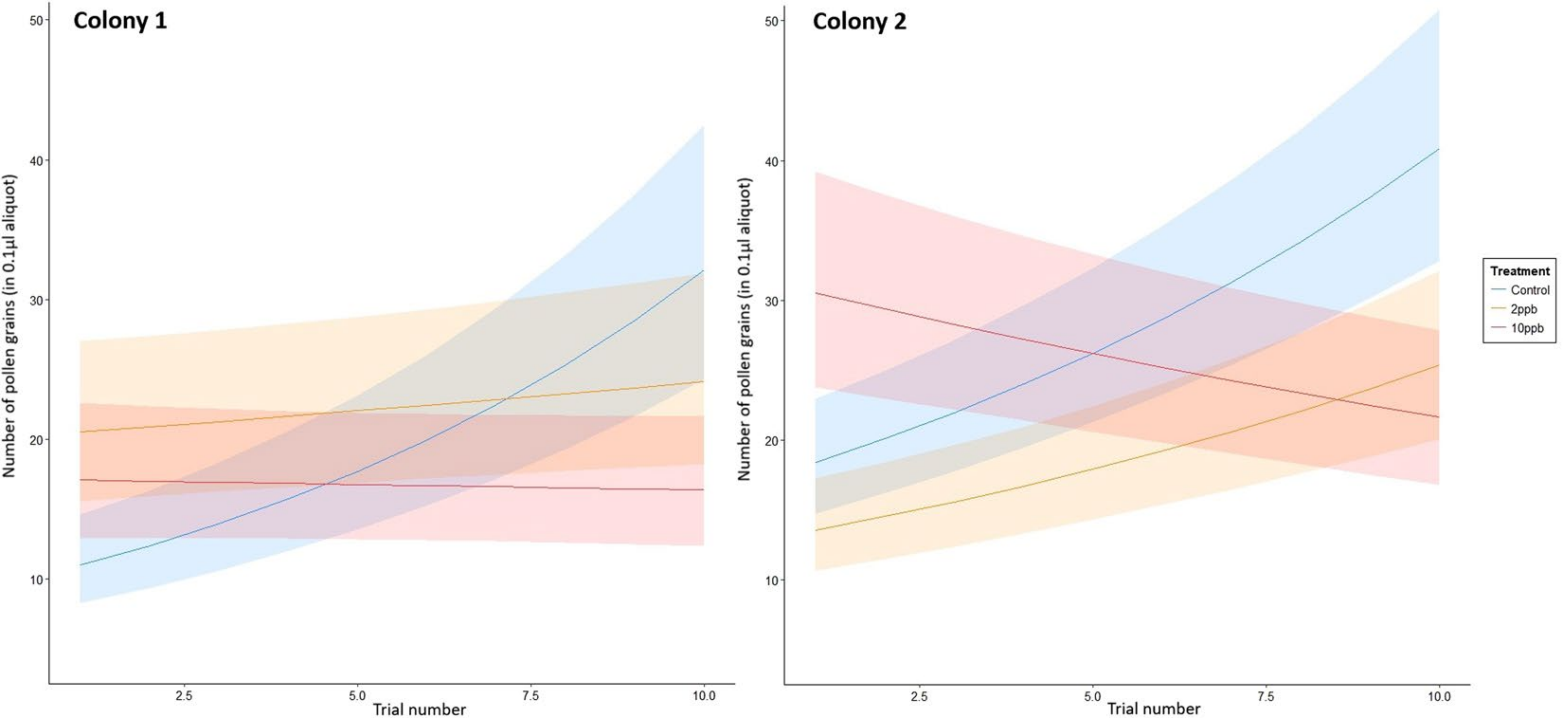
Model predictions from the Poisson GLMM for pollen collected in the three treatments. Lines represent mean values from model fits and shaded areas correspond to 95% confidence intervals.

Bees were observed to collect more pollen with increasing experience, but this ability was disrupted by exposure to thiamethoxam (trial by treatment interaction in colony 1: χ^2^_2_ = 93.43, p < 0.001; colony 2: χ^2^_2_ = 131.12, p < 0.001, Table 2, Fig. 1). By the tenth trial control bees were collecting an average of 95% and 126% more pollen than bees in the 2ppb group and 10ppb groups respectively (Fig. 1).

**Table 2:** Parameter estimates and 95% Confidence Intervals (CIs) from the Poisson GLMM for the number of pollen grains in 0.1 µl aliquot. The parameter estimates shown here are with reference to the control treatment group and are shown in the log link scale.

Pollen collection		Colony 1			Colony 2		
		Parameter Estimate	Lower 95% C.I.	Upper 95% C.I.	Parameter Estimate	Lower 95% C.I.	Upper 95% C.I.
Intercept		2.280	1.989	2.571	2.824	2.598	3.050
Treatment	2ppb	0.724	0.319	1.129	−0.286	−0.623	0.051
	10ppb	0.563	0.156	0.970	0.633	0.292	0.974
Trial		0.119	0.100	0.139	0.089	0.074	0.104
Treatment x trial	2ppb	−0.101	−0.127	−0.075	−0.019	−0.043	0.005
	10ppb	−0.124	−0.150	−0.098	−0.127	−0.150	−0.104

### Buzzing effort

**Figure 2:**
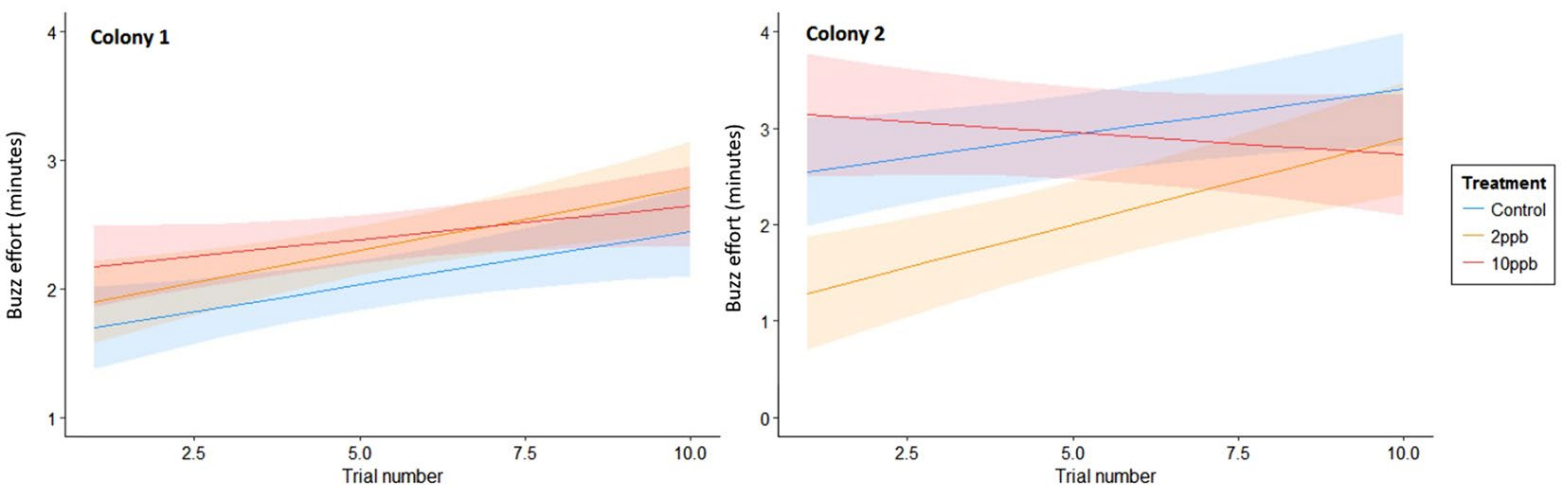
Model predictions from the linear mixed-effects model for buzzing effort across the three treatments. Shaded areas correspond to 95% confidence intervals.

Buzzing effort increased with experience and in Colony 1 there was no interaction with treatment, meaning that we found no evidence for different rates of change of buzzing effort among the treatment groups (χ^2^_2_ = 1.33, p = 0.515). Furthermore, in this colony, the 10ppb group had a greater overall buzzing effort than the control group (χ^2^_2_ = 6.81, p = 0.03, Fig. 2, Table 3). In contrast, the trial by treatment interaction was significant in Colony 2 (χ^2^_2_ = 12.03, p = 0.002), with the 10ppb group decreasing their buzzing effort with experience in contrast to the control and 2ppb groups (Fig. 2). In this colony the control and 2ppb groups increased their effort at similar rates but the 2ppb group had a lower overall buzzing effort (Table 3).

**Table 3:** Parameter estimates and 95% CIs from the linear mixed effect models for buzzing effort. The parameter estimates shown here are with reference to the control treatment group. Results in italics denote non-significant relationships.

Buzzing effort		Colony 1			Colony 2		
		Parameter Estimate	Lower 95% C.I.	Upper 95% C.I.	Parameter Estimate	Lower 95% C.I.	Upper 95% C.I.
Intercept		1.648	1.389	1.907	2.449	1.841	3.057
Treatment	2ppb	0.267	−0.002	0.536	−1.342	−2.228	−0.456
	10ppb	0.340	0.076	0.604	0.731	−0.193	1.655
Trial		0.077	0.043	0.111	0.096	0.012	0.180
Treatment x trial	2ppb	*0.017*	*−0.067*	*0.101*	0.083	−0.037	0.203
	10ppb	*−0.030*	*−0.114*	*0.054*	−0.144	−0.269	−0.019

### Buzz duration

**Figure 3:**
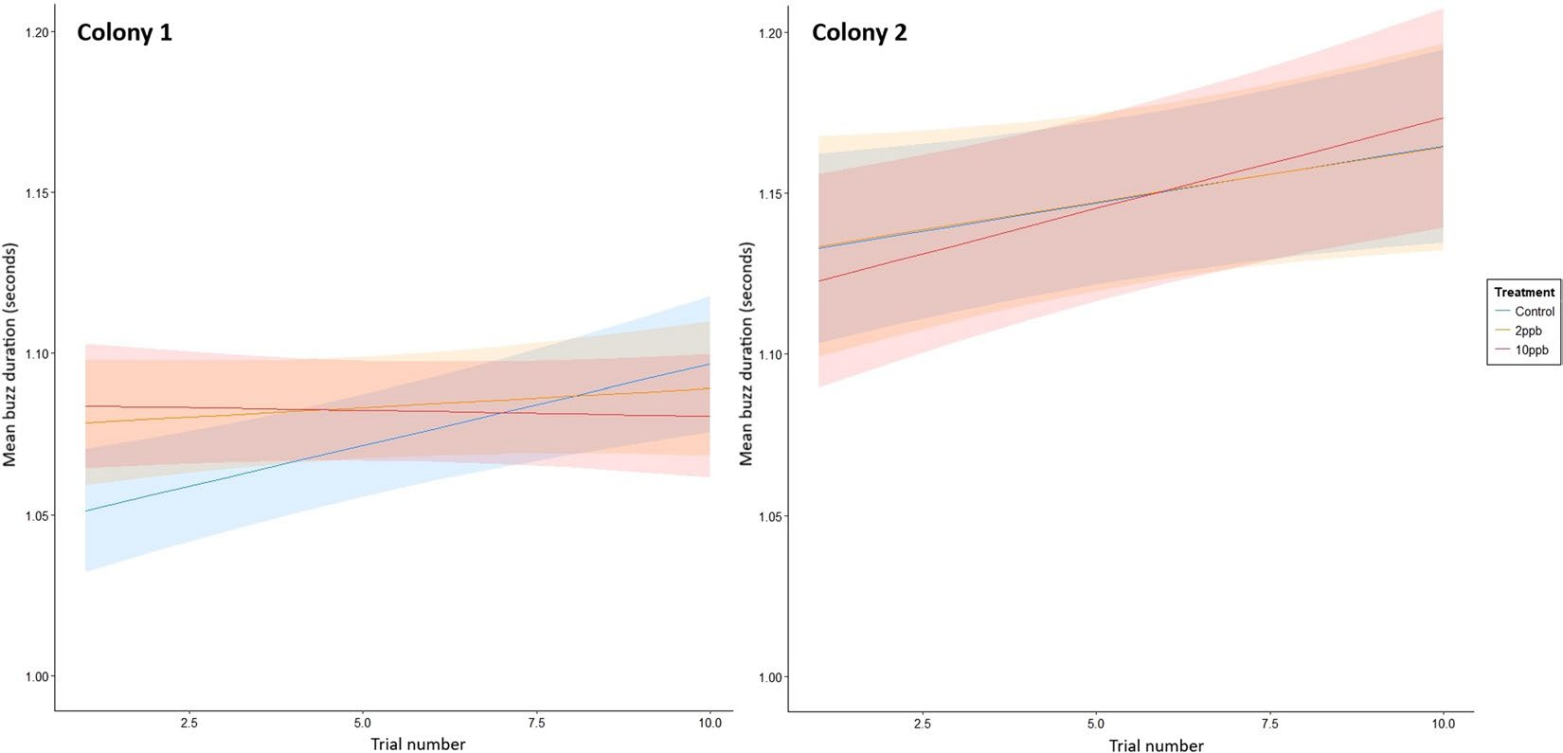
Model predictions from the linear mixed-effects model for mean buzz duration (Box-Cox transformed) across the three treatments. Shaded areas correspond to 95% confidence intervals.

Mean buzz duration increased with experience and, in contrast to the result for buzzing effort, there was an interaction between treatment and trial for Colony 1 (χ^2^_2_ = 8.46, p = 0.015) but not for Colony 2 (χ^2^_2_ = 0.79, p = 0.675). In Colony 1 the treated bees did not increase their mean buzz duration to the same extent as the control bees but in Colony 2 all groups increased their buzz duration at similar rates (Fig. 3, Table 4).

**Table 4:** Parameter estimates and 95% CIs from the linear mixed effect models for mean buzz duration. The parameter estimates shown here are with reference to the control treatment group. Results in italics denote non-significant relationships.

Buzz duration		Colony 1			Colony 2		
		Parameter Estimate	Lower 95% C.I.	Upper 95% C.I.	Parameter Estimate	Lower 95% C.I.	Upper 95% C.I.
Intercept		1.046	1.025	1.067	1.126	1.107	1.145
Treatment	2ppb	0.031	0.001	0.061	*−2.04E-04*	*−0.037*	*0.037*
	10ppb	0.038	0.009	0.067	*−0.001*	*−0.039*	*0.037*
Trial		0.005	0.002	0.008	0.004	0.002	0.006
Treatment x trial	2ppb	−0.004	−0.008	0.000	*−9.42E-05*	*−0.005*	*0.006*
	10ppb	−0.005	−0.009	−0.001	*0.002*	*−0.003*	*0.007*

### Acoustics of pollination and flight buzzes

**Figure 4:**
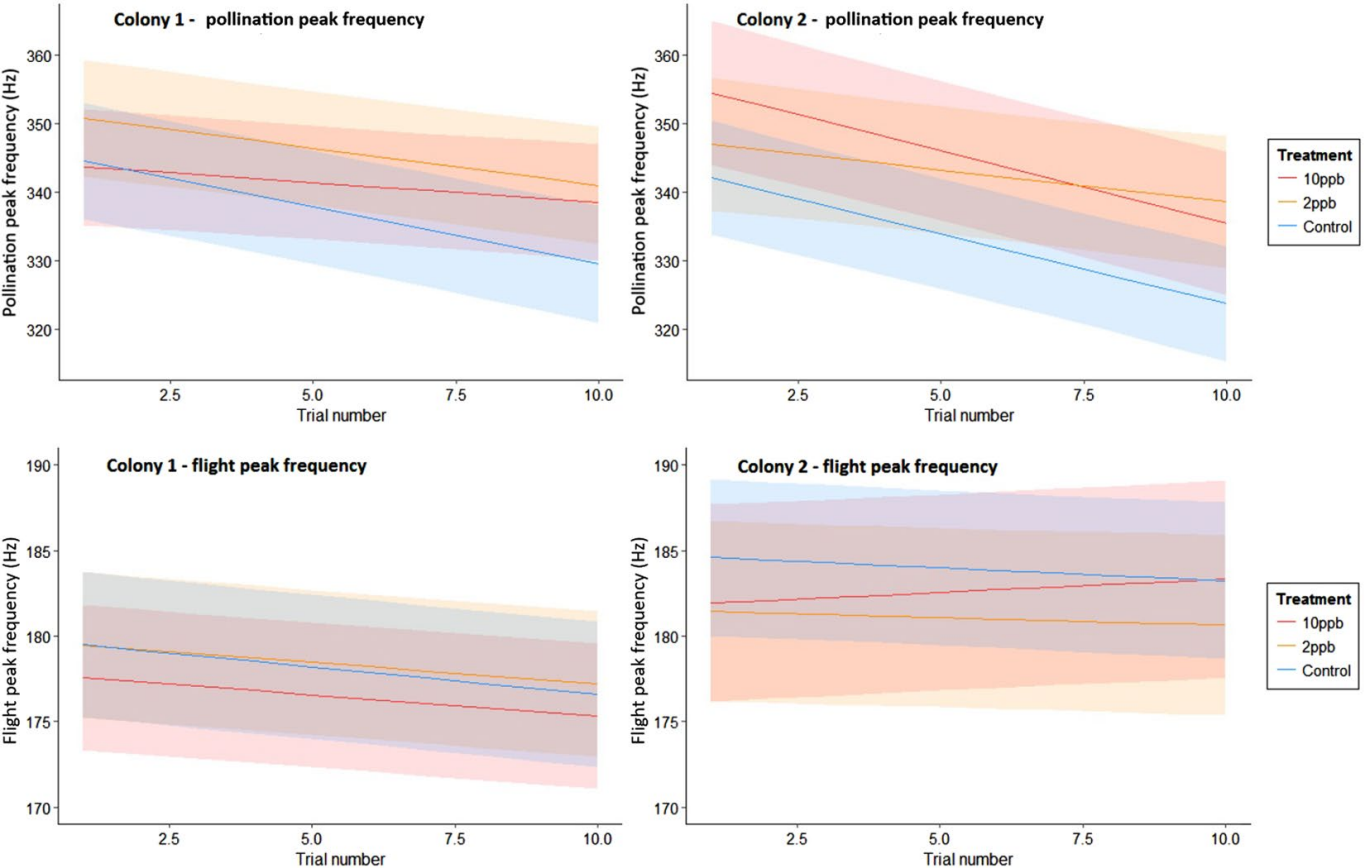
Model predictions from the linear mixed-effects model for peak frequency of the pollination and flight buzzes for both colonies across the three treatments. Shaded areas correspond to 95% confidence intervals.

The peak frequency of the pollination buzzes declined with increasing experience, but this occurred to a lesser extent in the 10ppb group of Colony 1 and in the 2ppb group of Colony 2 (trial by treatment interaction in Colony 1: χ^2^_2_ = 11.56, p = 0.003; Colony 2: χ^2^_2_ = 9.88, p = 0.007, Table 5, Fig. 4). The peak frequency of flight buzzes also declined over the course of the experiment, but to a lesser extent (Fig. 4). In Colony 2, this decline in flight peak frequencies was dependent on treatment because the 10ppb group did not show a reduction over time (χ^2^_2_ = 7.86, p = 0.02). Colony and treatment had no effect on the amplitude (dB) of pollination buzzes (χ^2^_1_ = 1.65, p = 0.200 & χ^2^_2_ = 0.58, p = 0.748 respectively). The amplitude of pollination buzzes did, however, significantly decline with increasing experience (χ^2^_1_ = 13.81, p < 0.001). The amplitude of the flight buzzes was also significantly affected by trial number, but in this case the amplitude increased over time (χ^2^_1_ = 10.39, p = 0.001). Again, colony and treatment had no effect on the amplitude of these buzzes (χ^2^_1_ = 0.81, p = 0.369 & χ^2^_2_ = 2.22, p = 0.330 respectively).

**Table 5:** Parameter estimates and 95% CIs from the linear mixed effect models for pollination peak frequency. The parameter estimates shown here are with reference to the control treatment group.

Peak frequency		Colony 1			Colony 2		
		Parameter Estimate	Lower 95% C.I.	Upper 95% C.I.	Parameter Estimate	Lower 95% C.I.	Upper 95% C.I.
Intercept		346.18	337.57	354.79	344.11	335.64	352.58
Treatment	2ppb	5.69	−6.50	17.88	3.81	−9.19	16.81
	10ppb	−1.94	−14.12	10.24	12.46	−1.18	26.10
Trial		−1.67	−2.13	−1.21	−2.04	−2.55	−1.53
Treatment x trial	2ppb	0.58	−0.08	1.24	1.10	0.33	1.88
	10ppb	1.10	0.47	1.73	−0.07	−0.87	0.73

## Discussion

We found that exposure to field realistic doses of the neonicotinoid thiamethoxam negatively impacted buzz pollination. Exposed bees showed less improvement in pollen collection with increasing experience than unexposed bees, and therefore collected 47% and 56% less pollen in the 2ppb and 10ppb groups respectively by the end of 10 trials (Fig. 1). This finding supports previous studies that have observed a reduced foraging efficiency in neonicotinoid exposed bees either by bringing back smaller pollen loads and/or foraging for pollen less often^10–12^. Improvement in pollen foraging performance over time has also been shown to be negatively impacted by exposure to imidacloprid^34^. This reduction in efficiency is thought to be due to altered interactions between bees and wildflowers^35^. Our study takes this research further and looks at both the interactions between bumblebees and flowers and also at how these interactions change over time. This offers further explanation for the negative impacts of neonicotinoids on foraging behaviour and pollination services.

Our findings suggest that the changes in bees’ abilities to buzz pollinate can arise in different ways, illuminating the complex nature of this form of pollination. It is already known that the peak amplitude and duration of sonication buzzes influences the amount of pollen released by *S. rostratum* flowers^27^, and that peak frequency declines with increasing experience, perhaps as part of a strategy to conserve energy^29^. We also found that the peak frequency of the pollination buzzes declined with experience but this was impacted by neonicotinoid exposure, with exposed bees in the 10ppb group of Colony 1 and in the 2ppb group of Colony 2 not reducing the frequency to the same extent. Further effects were, interestingly, colony-specific. Although both of the colonies used here responded to thiamethoxan exposure similarly in terms of the pollen they collected (Fig. 1), they responded quite differently in terms of buzz duration and effort (Figs 2 and 3). Mean buzz duration was impacted by treatment in Colony 1, with the control bees increasing the duration of their buzzes with increasing experience more than the treated bees (although this effect was marginal with the 2ppb group). This effect was not found in Colony 2, where treatment instead impacted the overall buzzing effort, with the control bees showing either an overall greater buzzing effort (than 2ppb-treated bees) or an increase in buzzing effort with experience (compared to a decrease, as in the case of 10ppb-treated bees).

Our findings therefore suggest that pesticide exposure impairs bees’ ability to improve pollen collecting ability as they gain experience. They may also indicate the existence of two distinct strategies for maximising pollen collection, as exemplified by these colonies: (1) extensions to the duration of buzzes (Colony 1); and (2) extensions of the overall time spent buzzing (Colony 2). Nevertheless, we found no indication that bees could overcome the apparent impact of exposure on their peak buzzing frequency. It is known that large inter-colony differences exist in bumblebees in life history traits such as colour preferences, learning and foraging performance^36,37^ but such marked differences in pollination strategies have not been previously noted, and represent a clear priority for further research.

Interestingly, in the early stages of our experiments the bees exposed to thiamethoxam appeared to make greater foraging efforts, particularly in Colony 1 where both treated groups had stronger buzzing efforts and longer buzz duration, ultimately collecting more pollen, in contrast to their performance at later stages. This is similar to findings in other experiments where bees exposed to thiamethoxam showed increased flower visitation rates^14,35^. This is thought to be a result of hormesis, where low doses of pesticide actually stimulate biological processes^38^ and has previously shown that other neonicotinoids can slightly improve learning and memory in honeybees^39^ and orientation behaviour in moths^40^. Our experiment has shown that although these effects might be initially observed, they show no benefit over a period of time in this instance.

It is unclear whether the reduction in pollen-collecting abilities found here would directly impact the fitness of the plant species that depend upon buzz pollination. However, reductions of this scale in the resources brought back to the colony by worker bees will stunt colony growth and impair the rearing of new queens (an effect of neonicotinoids previously reported^15^). Such a reduction in fitness is likely to have a negative impact on populations over larger temporal and spatial scales. Indeed, Woodcock *et al*.^5^ found evidence of increased population extinction rates in wild bees in response to neonicotinoid usage. This loss of pollinators is very likely to have detrimental effects for the crops and wild flowers that so depend on them. As a result, better understanding of the process of buzz pollination and its sensitivities to the impacts of neonicotinoid pesticides is an urgent requirement for sustainable agriculture.
